# The distribution of work-related musculoskeletal disorders among nurses in sub-Saharan Africa: a scoping review protocol

**DOI:** 10.1186/s13643-021-01774-7

**Published:** 2021-08-13

**Authors:** Kagiso Kgakge, Mbuzeleni Hlongwa, Themba Ginindza

**Affiliations:** 1 Discipline, of Public Health, School of Nursing and Public Health, University of KwaZulu-Natal, Durban, 4001 South Africa; 2 Burden of Disease Research Unit, South African Medical Research Council, Cape Town, South Africa

**Keywords:** Musculoskeletal disorders, Nurses, Low back ache, Hospitals, Sub-Saharan Africa, Prevalence, Incidence, Mortality, Risk factors, Economic costs

## Abstract

**Background:**

Although measures have been put in place, musculoskeletal injuries are noticeable high among the nursing fraternity with low back pain (LBP) being the most prevalent. It is evident that healthcare professionals are in constant exposure to occupational hazards such as musculoskeletal injuries as they discharge their professional duties. Not only does LBP affect the health of the nurses, it also creates a huge burden on the health systems with consequent poor performance at the workplace as well as economic burden. Therefore, the main objective of this study is to map evidence on the prevalence, incidence, mortality, risk factors, and economic costs of musculoskeletal disorders (MSD) in sub-Saharan Africa (SSA). This is a scoping review because we want to map the evidence of MSD among nurses in SSA and to identify the scope of body literature in which the findings will be used for planning the intervention study thereafter.

**Methods and analysis:**

Scoping review will be done to explore, describe, and map literature on the prevalence, incidence, mortality, risk factors, and economic costs related to MSD among nurses in SSA. The search will be done using databases such as PubMed, EBSCOHOST, Scopus, Web of Science, Science direct, Sabinet, WorldCat Local (iCatalogue), MEDLINE, CINAHL, Google Scholar, nursing academic editions, and World Health Organization (WHO) library databases. The search will look for primary studies within peer-reviewed articles as well as gray literature. In addition, the researcher will search for articles using keywords from the included studies as well as the list of references for related studies. The screening will be guided by Arksey and O’Malley’s framework which has five steps to be followed: (I) identifying the research question, (II) identifying relevant studies, (III) study selection, (IV) charting the data, and (V) collating, summarizing, and reporting the results, and the scoping review will be reported in accordance with the Preferred Reporting Items for Systematic reviews and Meta-Analyses extension for Scoping Reviews (PRISMA-ScR) checklist. A thematic content analysis will be used to give the narrative account of the review; using NVivo version 11 software, two independent reviewers will follow the three stages outlined by the thematic synthesis theory: (a) coding the findings of the included studies line-by-line, (b) organizing these free codes into related areas to construct descriptive themes, and (c) developing analytical themes. The outcome of coding will be verified and discussed with a third reviewer. The process of cross-checking the outcomes of coding of each included article will be discussed thoroughly.

**Discussion:**

At the end, this study anticipates to uncover the relevant literature in SSA in regard to the prevalence, incidence, mortality, risk factors, and economic costs related to MSD among nurses; furthermore, findings from this study will help in identifying research gaps; informing policy, priority in funding, and planning; and guiding future research.

**Supplementary Information:**

The online version contains supplementary material available at 10.1186/s13643-021-01774-7.

## Background

Work-related musculoskeletal disorders (WMSD) are described as musculoskeletal disorders that arise from work-related events [1]. On the other hand, musculoskeletal disorders (MSD) are defined as conditions that include a gamut of inflammatory and degenerative conditions that affect the tendons, muscles, joints, ligaments, peripheral nerves, and supporting blood vessels with consequent pain, ache, or discomfort [2]. Literature indicates that MSD have increasingly become a major public health problem and a significant leading occupational burden among nurses globally.

Epidemiological studies revealed that MSD are a major occupational health problem in the nursing fraternity due to the nature of its job [3]. A number of factors have been related to the development of MSD among nurses: physical factors like manual handling of patients and long-standing hours; psychosocial factors like stress, anxiety, and depression; and organizational factors like working shifts, shortage of staff, and poor working conditions [4, 5]. Therefore, MSD pose a major threat to the quality of life for nurses, resulting in work absenteeism, work limitations, and ultimate need to change jobs [6].

Research findings reveal a noticeable high prevalence of LBP being the most reported MSD among nurses ranging from 33 to 90.1% globally. Although LBP is regarded as an insignificant condition especially in SSA, the Global Burden of Disease (GBD 2015) has indicated that LBP is the leading cause of disability associated with a significant amount of cost. Furthermore, limited research around this field has prevailed with more attention focusing on epidemic infectious diseases such as HIV/AIDS, TB, and malaria in the African region [7].

Despite numerous efforts and measures that have been put in place to help curb the prevalence of MSD among nurses, the prevalence of MSD still prevails. It is therefore possible that the findings to this study will address this knowledge gap with documented evidence on the burden of MSD among nurses in SSA as well as advocate for sound strategies to tackle the disorder like governments coming up with ergonomic interventions such as zero lifting policy, exercise programs, and regular assessment of workplaces at regular intervals by safety and health professionals to ensure that all necessary preventative measures are implemented to alleviate the impact of the disease to the nurses.

Therefore, this scoping review will seek to find the evidence on prevalence, incidence, mortality, associated risk factors, and economic costs on MSD among nurses with the aim of developing interventions directed at controlling and preventing the development of MSD among nurses in SSA, with further emphasis on change of the research focus and funding directions in this field.

## Methods and materials

The scoping review will be reported in accordance with the Preferred Reporting Items for Systematic reviews and Meta-Analyses extension for Scoping Reviews (PRISMA-ScR) checklist [8].

### Scoping review framework

The proposed framework by Arksey and O’Malley will be adopted for this scoping review [9]. The framework involves (I) identifying the research question, (II) identifying relevant studies, (III) study selection, (IV) charting the data, and (V) collating, summarizing, and reporting the results.

#### Identifying the research question

The main research question is: “What is the evidence on the burden of work-related musculoskeletal disorders in Sub-Saharan Africa?”.

Research sub-questions will be as follows:

- What is the burden of WMSD among nurses in sub-Saharan Africa with estimation in relation to prevalence, incidence, and mortality?
- What are the risk factors associated with WMSD?
- What are the economic costs associated with WMSD?

##### Eligibility criteria

Studies will be selected according to the population, exposure, and outcomes (PEO) framework outlined below (Table 1).

**Table 1:** The PEO framework for eligibility of research question

Criteria	Determinants
Population and their problem	Nurses with MSD in sub-Saharan Africa
Exposure	Hospital work
Outcome	1. Prevalence
	2. Incidence
	3. Mortality
	4. Risk factors
	5. Economic costs
	6. Low back ache

#### Identifying relevant studies

Keyword search will be conducted from various electronic databases without a date limit: PubMed, CINAHL, Google Scholar, nursing academic editions and World Health Organization (WHO) library databases, Ebscohost, Scopus, Science direct, Scopus, Web of Science, Sabinet via SA ePublications, and WorldCat Local (iCatalogue). Furthermore, we will work closely with the University of KwaZulu-Natal library services during the database searching and retrieval to improve the quality of our search strategy of articles. Keywords that will be used to search for these databases are as follows: work related musculoskeletal disorders, nurses, musculoskeletal disorders, low back ache, prevalence, incidence, mortality, economic costs related to MSD, and risk factors. During the search, keywords will be separated by Boolean terms (AND, OR). MESH terms will also be used (Appendix 1). Furthermore, the reference lists of studies eligible for inclusion will be screened for potential additional articles. Studies obtained through database searches will be exported to Endnote library for further abstract and full article screening respectively. The Endnote library “find full text” option will be used to automatically download PDFs of exported studies. Due to under-representation of literature in this area, we will do a global search approach then contextual interpretation for sub-Saharan Africa.

#### Study selection

Two independent reviewers will conduct screening, guided by the eligibility criteria for this review. A broad database search will initially be conducted against broad inclusion criteria by the first reviewer (KK). All identified articles that will be potentially eligible for this study will be obtained in full texts; thereafter, two independent reviewers (KK and MH) will conduct abstracts and full article screenings to identify articles that meet all the inclusion criteria. The support from the University of KwaZulu-Natal library services will be sought during database searching and retrieval of articles. The Preferred Reporting Items for Systematic reviews and Meta-Analyses extensions for Scoping Reviews (PRISMA-ScR) flow diagram will be used to document the review process [8]. Whenever there is a disagreement between the two reviewers, it will be resolved through consensus and will involve a third reviewer.

##### Eligibility criteria

Inclusion criteria

- Study will include nurses with low back ache
- All the study designs with relevant intervention
- Studies that focus on work-related musculoskeletal disorders among nurses; prevalence, incidence, mortality, economic costs, and risk factors
- Only studies conducted in English and in other languages with an English version will be included

Exclusion criteria

- Studies that do not focus on nurses and low back ache
- Studies that do not focus on work-related musculoskeletal disorders among nurses
- Studies published in languages other than English and do not have an English version will also be excluded

PRISMA flow chart will be used to report the screening results.

PRISMA flow chart (Additional file 1: Figure S1) will be used to summarize the inclusion and exclusion criteria.

#### Charting the data

The relevant data will be drawn out using an extraction form (Table 3 in Appendix 2). In addition to answering the research question, a charting form will be developed in such a way that it covers all the needed variables and it will be regularly updated when the need arises. The extraction form will continually be updated. Then, the form (Table 2) which includes the following: author with date, study title, study design, study setting, population, study aim, intervention, percentages, outcomes of the study, key findings, and comments will be used to chart the data of all the reviewed articles, coded using a coding system. This is purposively done to keep track of the studies included and excluded during the charting process of the scoping review. Two independent reviewers (KK and MH) will do data charting by following the three stages outlined by the thematic synthesis theory: (a) coding the findings of the included studies line-by-line, (b) organizing these free codes into related areas to construct descriptive themes, and (c) developing analytical themes.

**Table 2:** Data charting form

Author and date
Study design
Study setting
Population
• Average age
• Sample size
Aims
Intervention
Outcome
Key findings
Conclusions
Comment

#### Collating, summarizing, and reporting the results

The proposed scoping review aims to map the existing evidence on the distribution of work-related musculoskeletal disorders among nurses in sub-Saharan Africa and to sum up the findings as presented in the relevant articles. All data relating to the prevalence of MSD and low back ache, incidence of MSD, mortality of MSD, risk factors of MSD, and economic costs related to MSD will be extracted and structured for the purpose of identifying themes. A descriptive report of the findings from the literature will be presented through thematic content analysis. The structure of the literature will assume the following estimates: prevalence, incidence, mortality, economic cost, and risk factors. The explanation of the findings will be analyzed to see how they relate to the overall study purpose and the implications of these findings for future research, policy, and practice.

##### Quality assessment

Assessment of the evidence from different studies will be conducted for quality; this will be done in order to ensure the appropriateness of the study design to the research objectives and to minimize the risk of bias. Methodological rigor will be achieved by having two independent reviewers critically appraising the methodological validity of the included studies. A quality appraisal tool which focuses on the study methods, the Mixed Method Quality Appraisal Tool (MMAT) version 2018, will be adopted and used [10]. MMAT is a critical appraisal tool which has been designed for the appraisal stage of systematic mixed studies reviews, that is, reviews that include qualitative, quantitative, and mixed methods studies. The tool helps users to appraise the methodological quality of five categories in studies: (a) qualitative research, (b) randomized controlled trials, (c) non-randomized studies, (d) quantitative descriptive studies, and (e) mixed methods studies. Furthermore, the tool will be used to examine the quality of an article looking at the following aspects: the appropriateness of the aim of the study, adequacy and methodology, study design, participant recruitment, data collection, data analysis, presentation of findings, authors’ discussions, and conclusions. If the response to the initial screening is either “no” or “can’t tell,” they will be excluded from further screening. For articles that will score below 50% on the methodological quality assessment, they will be excluded to ensure that we only include studies with a strong methodological rigor to answer the study’s research question.

## Discussion

Work-related musculoskeletal disorders (WMSD) have emerged to be a major public health concern globally; therefore, the proposed scoping review seeks to map existing evidence regarding the distribution (prevalence, incidence, and mortality), risk factors, and estimated costs associated to MSD among nurses in SSA in order to publish the research gaps in this area which has sparse data. According to [2], MSD are the most common cause of morbidity among healthcare workers especially nurses. The high prevalence of MSD among nurses has been linked to the physical nature of their job and the organizational factors by many researchers [1–5]. The burden of MSD among nurses is commonly associated with a number of factors such as lifting patients, lifting heavy loads, patient transfer from the floor and out of bed, and working in awkward postures resulting in acute and accumulative MSD.

This study will map the evidence on the distribution of MSD with emphasis on the trends of low back pain (LBP) among nurses. Most studies that have been conducted on MSD among nurses indicate that LBP is the most prevalent and a major cause of disability affecting the general well-being and performance. The 2010 Global Burden of Disease Study estimated that LBP is among the top 10 diseases and injuries that account for the highest number of disability-adjusted life years (DALY) worldwide resulting in activity limitation and work absence with a consequent high economic burden on individuals, families, communities, industry, and governments [11].

It is critical to map the evidence of MSD among nurses as this will bring out more understanding towards this disease with the aim of directing more efforts towards the health of healthcare workers, policy makers, and all relevant stakeholders to fight this scourge. Furthermore, this review will uproot an economic projection of LBP and a coherent healthcare resource allocation as well as intervention program towards MSD in order to minimize the severity and impact on healthcare workers.

## Conclusion

Findings from this proposed scoping review are expected to give a reflection on the distribution of MSD among nurses in the SSA region with estimates on prevalence, incidence, mortality with the identification of risk factors, and the associated economic burden. Therefore, the evidence synthesized from this study will help researchers, decision-makers, and other stakeholders to inform policy and ensure an efficient allocation of healthcare resources, improving the healthcare system performance and thus improving treatment and reducing morbidity and mortality associated with MSD.

### Supplementary Information

**Additional file 1: Figure 1.** PRISMA flow diagram.

## Data Availability

All data generated or analyzed during this study will be included in the published systematic review article.

## Appendix 1

### Draft search strategy

((((((("musculoskeletal diseases"[MeSH Terms] OR ("musculoskeletal"[All Fields] AND "diseases"[All Fields]) OR "musculoskeletal diseases"[All Fields] OR ("musculoskeletal"[All Fields] AND "disorders"[All Fields]) OR "musculoskeletal disorders"[All Fields]) AND ("nurses"[MeSH Terms] OR "nurses"[All Fields])) AND prevalance[All Fields]) OR ("risk factors"[MeSH Terms] OR ("risk"[All Fields] AND "factors"[All Fields]) OR "risk factors"[All Fields])) AND ("epidemiology"[Subheading] OR "epidemiology"[All Fields] OR "incidence"[All Fields] OR "incidence"[MeSH Terms])) AND (("economics"[MeSH Terms] OR "economics"[All Fields] OR "economic"[All Fields]) AND ("costs and cost analysis"[MeSH Terms] OR ("costs"[All Fields] AND "cost"[All Fields] AND "analysis"[All Fields]) OR "costs and cost analysis"[All Fields] OR "costs"[All Fields]))) AND ("mortality"[Subheading] OR "mortality"[All Fields] OR "mortality"[MeSH Terms])) AND (("economics"[MeSH Terms] OR "economics"[All Fields] OR "economic"[All Fields]) AND ("costs and cost analysis"[MeSH Terms] OR ("costs"[All Fields] AND "cost"[All Fields] AND "analysis"[All Fields]) OR "costs and cost analysis"[All Fields] OR "costs"[All Fields])).

## Appendix 2

Table

**Table 3:** Data extraction form

Author and date of publication Study title Aim(s) of the study or research questions Study design
Study setting (urban/rural)
Population
- Average age
- Gender
- Sample size
- Percentage of women
- Percentage of men
Methodology/intervention Type of Intervention and outcomes
Most relevant finding Most significant finding
Other key findings Study limitations and implications
Interpretations and conclusions from the authors
Comment/s

## Abbreviations

DALY: Disability-adjusted life year
GBD: Global Burden of Disease
LBP: Low back pain
MMAT: Mixed Method Quality Appraisal Tool
MSD: Musculoskeletal disorders
PRISMA-P: Preferred Reporting Items for Systematic Reviews and Meta-Analysis Protocols
SSA: Sub-Saharan Africa
WHO: World Health Organization
WMSD: Work-related musculoskeletal disorders

## References

[1] Salik Y, Özcan A (2004). Work-related musculoskeletal disorders: a survey of physical therapists in Izmir-Turkey. BMC Musculoskelet Disord.

[2] Punnett L, Wegman DH (2004). Work-related musculoskeletal disorders: the epidemiologic evidence and the debate. J Electromyogr Kinesiol.

[3] Burdorf A, Sorock G (1997). Positive and negative evidence of risk factors for back disorders. Scand J Work Environ Heal.

[4] Rathore FA, Attique R, Asmaa Y (2017). Prevalence and perceptions of musculoskeletal disorders among hospital nurses in Pakistan: a cross-sectional survey. Cureus.

[5] Tinubu BM, Mbada CE, Oyeyemi AL, Fabunmi AA (2010). Work-related musculoskeletal disorders among nurses in Ibadan, South-west Nigeria: a cross-sectional survey. BMC Musculoskelet Disord.

[6] Choi SD, Brings K (2016). Work-related musculoskeletal risks associated with nurses and nursing assistants handling overweight and obese patients: a literature review. Work.

[7] Kahere M, Ginindza T (2020). Mapping evidence on the prevalence, incidence, risk factors and cost associated with chronic low back pain among adults in Sub-Saharan Africa: a systematic scoping review protocol. Syst Rev.

[8] McGowan J (2020). Reporting scoping reviews-PRISMA ScR extension. J Clin Epidemiol.

[9] Arksey H, O’Malley L (2005). Scoping studies: towards a methodological framework. Int J Soc Res Methodol Theory Pract.

[10] Hong QN, Pluye P, Fàbregues S, Bartlett G, Boardman F, Cargo M, et al. Mixed methods appraisal tool (MMAT), version 2018. Canada: Canadian Intellectual Property Office; 2018. http://mixedmethodsappraisaltoolpublic.pbworks.com/w/file/fetch/127425851/MMAT_2018_criteria-manual_2018-04-04.pdf. Accessed 12 Sept 2020.

[11] Coates MM, Kintu A, Gupta N, Wroe EB, Adler AJ, Kwan GF, Park PH, Rajbhandari R, Byrne AL, Casey DC, Bukhman G. Burden of noncommunicable diseases from infectious causes in 2017: a modelling study. Lancet Glob Health. 2020;8(12):e1489–e1498. 10.1016/S2214-109X(20)30358-2. 10.1016/S2214-109X(20)30358-2 PMC8040338 33098769

