# Supplementary figures and images for: The distribution of work-related musculoskeletal disorders among nurses in sub-Saharan Africa: a scoping review protocol
Source: Syst Rev. 2021 Aug 13;10:229. doi: 10.1186/s13643-021-01774-7 (PMC8364119; doi:10.1186/s13643-021-01774-7)

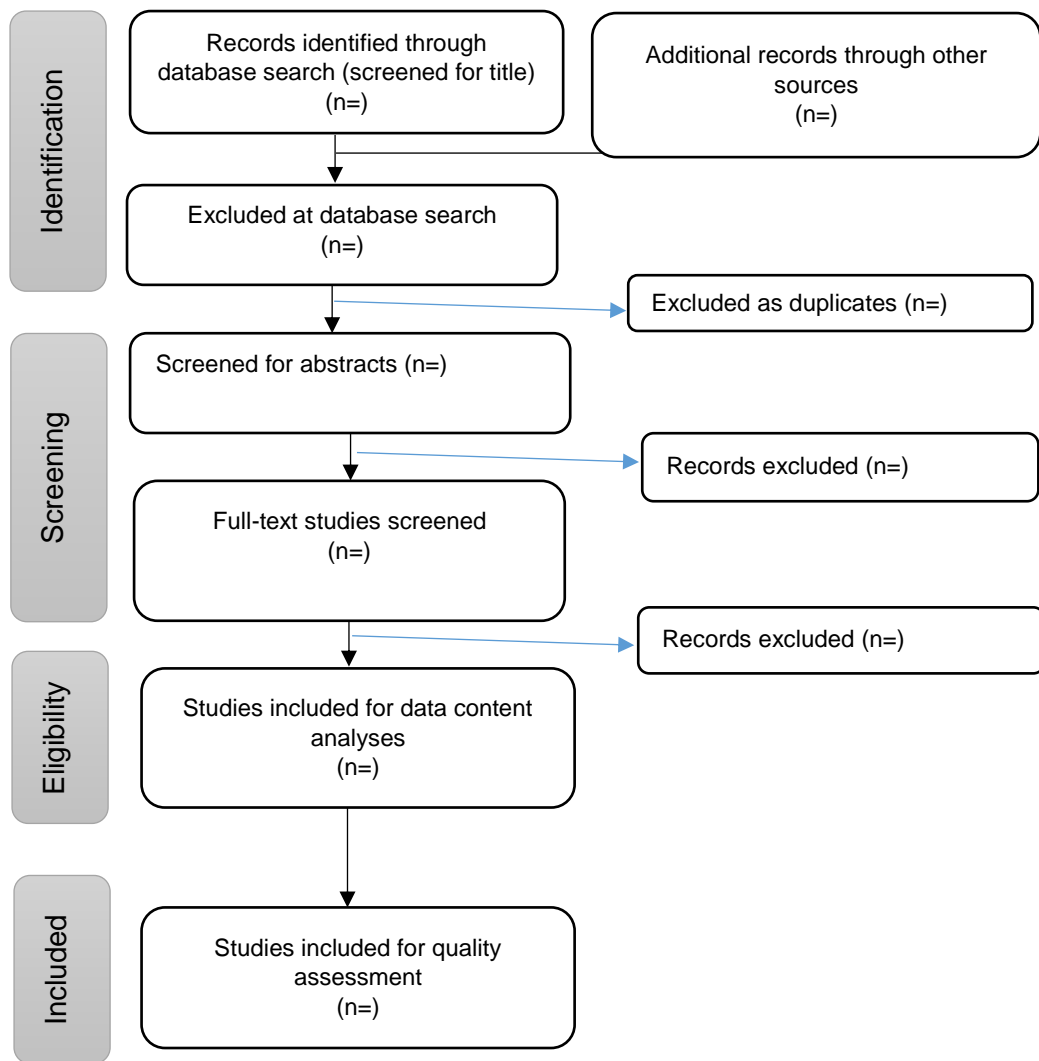

**Figure 1:** PRISMA flow diagram

Supplement: Supplementary file 1 — Additional file 1: Figure 1. PRISMA flow diagram. [file 13643_2021_1774_MOESM1_ESM.pdf]
